# Lower-Limb Muscle Architecture and Strength in Men with Radiographic Axial Spondyloarthritis: A Cross-Sectional Ultrasound Study

**DOI:** 10.3390/jcm15197515

**Published:** 2026-09-27

**Authors:** Mehmet Serkan Kılıçoğlu, Mert Kara, Esra Kültür Çamlı, Delal Lutfi, Buğra İnce, Teoman Aydın, Ozan Volkan Yurdakul

**Affiliations:** 1 Department of Physical Medicine and Rehabilitation, Faculty of Medicine, Bezmialem Vakıf University, Adnan Menderes Boulevard, Istanbul 34093, Türkiye; 2 Physical Medicine and Rehabilitation Clinic, Özel Yaşam Hastanesi, Antalya 07160, Türkiye; 3 Department of Physical Medicine and Rehabilitation, Kütahya City Hospital, Kütahya 43100, Türkiye; 4 Department of Physical Medicine and Rehabilitation, İzmir Faculty of Medicine, University of Health Sciences Türkiye, İzmir City Hospital, İzmir 35540, Türkiye

**Keywords:** radiographic axial spondyloarthritis, muscle ultrasound, muscle architecture, pennation angle, muscle thickness, muscle strength, C-reactive protein

## Abstract

**Background/Objectives**: To compare lower-limb muscle architecture and strength between men with radiographic axial spondyloarthritis (r-axSpA) and controls. **Methods**: In this controlled cross-sectional study, 30 men with r-axSpA and 30 age-matched healthy men underwent bilateral ultrasound assessment of the vastus lateralis (VL), gastrocnemius medialis (GM), gastrocnemius lateralis (GL), and tibialis anterior (TA). Fascicle length was derived trigonometrically. Strength was assessed using hand-held dynamometry. **Results**: Differences represent r-axSpA minus controls, with 95% confidence intervals. VL, GM, and GL thickness differences were −0.32 (−0.50 to −0.13), −0.18 (−0.34 to −0.02), and −0.21 (−0.36 to −0.05) cm, respectively. Pennation-angle differences were −2.38 (−3.48 to −1.28), −1.99 (−3.35 to −0.63), −2.46 (−3.69 to −1.23), and 0.26 (−0.68 to 1.22) degrees for VL, GM, GL, and the TA superficial region, respectively. TA superficial-region thickness and derived fascicle length were greater: +0.53 (0.29 to 0.78) and +2.40 (1.36 to 3.45) cm, respectively. Knee extensor, ankle dorsiflexor, and ankle plantar flexor strength differences were −86.3 (−123.6 to −48.8), −52.0 (−78.2 to −26.6), and −57.9 (−92.3 to −24.1) N, respectively. **Conclusions**: Men with r-axSpA showed greater TA superficial-region thickness and derived fascicle length alongside lower dorsiflexion strength. These preliminary findings require confirmation in independent studies using validated measurement methods.

## 1. Introduction

Axial spondyloarthritis (axSpA) is a chronic inflammatory rheumatic disease characterized by inflammatory back pain, stiffness, and progressive restriction of spinal mobility [1]. Beyond the axial skeleton, several factors may contribute to peripheral muscle impairment. Lower objectively measured physical activity has been reported in axSpA compared with healthy controls [2], while high levels of kinesiophobia have been documented in ankylosing spondylitis [3]. Reduced lean mass has also been associated with higher disease activity, supporting a possible contribution of inflammatory disease burden [4]. Differences in gait parameters, including walking speed and step length, suggest altered movement patterns that may affect lower-limb loading [5]. These observations provide plausible explanations but do not establish the causal contribution of each factor to muscle architectural changes [6]. Imaging studies have documented atrophy and fatty infiltration of paravertebral muscles in established disease [7,8], whereas the lower-limb muscles that contribute to gait, balance, and functional mobility have been studied less extensively.

Muscle ultrasound is a portable, non-ionizing method for characterizing muscle size and architecture. Recommended ultrasound domains include muscle thickness, cross-sectional area, fascicle length, pennation angle, and echogenicity [9]. Muscle thickness and pennation angle are related to the arrangement and force-generating capacity of muscle fascicles, although architecture alone does not determine voluntary strength [10,11,12,13,14]. Standardized protocols can provide reliable measurements of limb muscles, including the vastus lateralis (VL) [15].

Peripheral muscle abnormalities have been described in other inflammatory rheumatic diseases. In rheumatoid arthritis, quadriceps morphology and activation have been linked with functional capacity [16], and a recent prospective muscle-ultrasound cohort showed that muscle morphology can change over time and may track inflammatory disease activity [17]. Recent studies have also directly examined sarcopenia and body composition in axSpA. Kanjanavaikoon et al. reported sarcopenia in 22.1% of 104 patients, with independent associations with older age, lower BMI, and greater functional limitation [18]. In a Spanish cohort of 94 patients aged ≥50 years, Berbel-Arcobé et al. reported sarcopenia in 3.2% and low muscle mass alone in 23.4%, using a different diagnostic framework [19]. These findings establish the relevance of muscle health in axSpA while illustrating variation across populations and assessment criteria. However, assessments of muscle mass and sarcopenia do not fully characterize muscle-specific pennation angles or fascicle geometry. The remaining question is how architecture differs across individual lower-limb muscles in radiographic axial spondyloarthritis (r-axSpA) and how these differences relate to strength in the corresponding joint actions.

The VL, gastrocnemius medialis (GM), gastrocnemius lateralis (GL), and tibialis anterior (TA) were selected to sample accessible superficial muscles contributing to knee extension, ankle plantar flexion, and ankle dorsiflexion. Their selection allowed assessment of muscle architecture across these functional groups using a focused bilateral ultrasound protocol. The gastrocnemius heads were selected rather than the deeper soleus to prioritize superficial imaging sites, while VL was selected as the quadriceps component rather than examining multiple quadriceps heads, including rectus femoris. This selection was not intended to provide a comprehensive assessment of all muscles contributing to the measured joint actions.

The primary objective of this study was to compare ultrasound-derived thickness, pennation angle, and fascicle length of the VL, GM, GL, and TA, together with corresponding isometric muscle strength, between men with r-axSpA and age-matched healthy men. For TA, architectural measurements represented the superficial region. Secondary objectives were to examine architecture–strength correlations, measurement reliability, differences according to biologic treatment status, and exploratory associations between muscle parameters and serum C-reactive protein (CRP). We hypothesized that men with r-axSpA would exhibit differences in lower-limb muscle thickness, pennation angle, and derived fascicle length, together with lower isometric strength, compared with healthy controls. Associations between muscle architecture and CRP were explored as secondary, hypothesis-generating analyses.

## 2. Materials and Methods

### 2.1. Study Design, Setting, and Ethics

This single-center controlled cross-sectional study prospectively enrolled participants between 28 January and 2 March 2024 at the Department of Physical Medicine and Rehabilitation, Bezmialem Vakıf University. Reporting follows the Strengthening the Reporting of Observational Studies in Epidemiology (STROBE) statement [20]. The Bezmialem Vakıf University Non-Interventional Clinical Research Ethics Committee approved the protocol (Protocol No. 2022/340; 15 November 2022; Meeting No. 24). All participants provided written informed consent. The study was submitted to ClinicalTrials.gov on 18 January 2024 and first publicly posted on 26 January 2024 (NCT06226792), before enrollment of the first participant on 28 January 2024. The registry’s study-start field was inadvertently entered as 18 January 2024, corresponding to the registration submission date rather than the actual first-enrollment date. The study was conducted in accordance with the Declaration of Helsinki.

### 2.2. Participants

Thirty-five consecutive men aged ≥18 years with r-axSpA were screened in a university outpatient clinic. Eligibility was restricted to men to reduce heterogeneity related to sex-associated differences in muscle architecture and strength within the planned sample. This restriction limits the applicability of the findings to men with r-axSpA. Patients with an established diagnosis of ankylosing spondylitis according to the modified New York criteria were included and are described here as having radiographic axial spondyloarthritis (r-axSpA) [21]. Radiographic sacroiliitis grades were obtained from existing clinical radiology reports. Radiographs were not independently re-read for this study; therefore, study-specific inter-rater reliability for radiographic grading was not assessed. Five eligible patients declined participation, leaving 30 patients. Thirty age-matched healthy male volunteers who responded to a study advertisement displayed in our outpatient clinic were enrolled as controls (Figure 1).

Exclusion criteria were a neuromuscular or metabolic disorder, advanced cardiovascular disease, participation in a regular exercise program, inability to walk or flex the knee, previous knee arthroplasty, juvenile disease onset, or refusal to consent. Hip joint involvement and peripheral arthritis were additional exclusion criteria; neither was present in the included patients. For this study, regular exercise was operationally defined as self-reported exercise for at least 30 min per session on at least three days per week. Individuals meeting this criterion were excluded from both groups. Exercise participation was assessed by interview only; no validated physical activity questionnaire or device-based assessment was used. The same general medical and mobility exclusions were applied to controls. No participant was excluded after enrollment.

### 2.3. Clinical and Functional Assessments

Age, height, weight, body mass index (BMI), educational level, limb dominance, symptom duration, time since diagnosis, medication use, and serum CRP were recorded. Disease activity was assessed with the Bath Ankylosing Spondylitis Disease Activity Index (BASDAI) [22]. Physical function and spinal mobility were assessed with the Bath Ankylosing Spondylitis Functional Index (BASFI) [23] and Bath Ankylosing Spondylitis Metrology Index (BASMI) [24], respectively. Higher BASDAI and BASFI scores indicate worse disease activity and function; higher BASMI scores indicate greater restriction of spinal mobility. The Achilles and patellar tendon insertion sites were assessed clinically and by ultrasound for enthesitis or enthesopathic changes. HLA-B27 status, radiographic sacroiliitis grades, smoking history, and cumulative duration of biological treatment were also recorded. Spinal structural damage was not scored using the modified Stoke Ankylosing Spondylitis Spinal Score (mSASSS).

### 2.4. Muscle Ultrasound

Bilateral VL, GM, GL, and TA were examined in B-mode using a LOGIQ P5 ultrasound system and an 8–10 MHz linear-array transducer (GE Healthcare, Chicago, IL, USA). A standardized initial setup was used, with adjustments to accommodate individual anatomy. Imaging depth was initially set to 4.0 cm and adjusted between 3.0 and 6.0 cm according to subcutaneous tissue thickness and muscle depth to include the anatomical boundaries required for the measurements within the field of view. One or two focal zones were positioned at the target muscle region and/or the aponeurosis used for measurement. Overall gain was initially set to 50%, with a flat time-gain compensation (TGC) profile. Minor adjustments to gain and TGC were made during acquisition to optimize visualization of the aponeuroses and fascicles while avoiding excessive image brightness and near-field saturation. Participants were instructed to keep the examined muscles relaxed during ultrasound acquisition. For VL imaging, participants lay supine with the hip at 0° flexion, verified using a goniometer, and the knee fully extended. No support was placed beneath the hip, thigh, or knee. The ankle was positioned at 90° relative to the lower leg, also verified using a goniometer, and maintained manually by an examiner as part of the standardized limb-positioning protocol, rather than to control VL muscle length. For GM and GL imaging, participants lay prone with the knee fully extended and the foot hanging freely beyond the edge of the examination table. For TA imaging, participants lay supine with the knee fully extended and the ankle positioned at 90°, verified using a goniometer and maintained manually by an examiner, without an external positioning device. The VL measurement site was located at 50% of the distance between the greater trochanter and the lateral knee joint line [25]. The GM site was located at one-third (approximately 33.3%) of the distance from the medial knee joint line to the medial malleolus. The GL site was located at one-third of the distance from the lateral knee joint line to the lateral malleolus. The TA site was located at one-third of the distance from the lateral tibial condyle to the lateral malleolus. All proportional distances were measured from the proximal landmark. Standardized participant positions and probe locations are illustrated in Figure 2.

Measurement sites were marked on the skin, and the same markings were retained for both examiners’ assessments. The second examiner did not independently re-identify the anatomical measurement sites. A generous amount of ultrasound gel was used, and transducer pressure was adjusted manually while monitoring the image for visible tissue compression. The transducer was aligned longitudinally to visualize fascicles and the relevant aponeurotic boundaries, including the superficial and central aponeuroses for TA. Probe force was not quantified using a pressure or force sensor; pressure control was based on the examiner’s visual assessment.

Two physiatrists with eight and four years of muscle-ultrasound experience each independently acquired three longitudinal images per muscle and side, yielding six images in total per muscle and side for each participant. For every acquisition, the examiner lifted the probe from the skin and repositioned it at the previously marked anatomical site before obtaining a new image.

Both examiners were blinded to group allocation and clinical and radiographic data during acquisition and measurement. Image quality was assessed in real time before freezing and saving each image. Probe orientation and contact pressure were adjusted until predefined technical criteria were met: appropriate aponeurosis alignment, continuity of the visualized fascicle segment, and absence of visible tissue compression. These criteria required visual judgment. No saved images were subsequently excluded from analysis. Individual probe adjustments and unsaved views were not logged; therefore, group-specific counts of rejected views were unavailable.

Saved images were de-identified and coded for offline measurement. Each examiner measured only the images that they had personally acquired; the second examiner did not measure images acquired by the first examiner.

For VL, GM, and GL, muscle thickness was measured as the perpendicular distance between the superficial and deep aponeuroses, and pennation angle was measured between a clearly visualized fascicle and the deep aponeurosis (Figure 3). For TA, thickness was measured as the perpendicular distance between the superficial and central aponeuroses, representing the superficial region rather than the full muscle thickness. TA pennation angle was measured between a fascicle within this superficial region and the central aponeurosis. The same anatomical boundaries were used in all participants in both groups; TA thickness measurements were not extended to the interosseous membrane.

Because the complete fascicle was not visible within the field of view, fascicle length was derived as Lf = MT/sin(θ). For TA, MT represented the perpendicular distance between the superficial and central aponeuroses, and θ represented the angle between a superficial-region fascicle and the central aponeurosis. Thus, the derived TA fascicle length represented the superficial region. Fascicle length was calculated separately for each image using the paired thickness and pennation-angle measurements from that image. For each parameter, an examiner-specific mean was calculated from that examiner’s three separately acquired images. The two examiner-specific means were then averaged for the primary analyses.

Intraobserver reliability was calculated separately for each examiner using measurements from their three separately acquired images per muscle and side. It therefore assessed within-session repeatability of the combined acquisition and measurement procedures, rather than repeated readings of the same stored image. Interobserver agreement compared the two examiner-specific means and reflected both acquisition and measurement variability. Because both examiners used the same skin markings, independent measurement-site localization was not assessed. Echogenicity was not analyzed because acquisition settings were not standardized for quantitative grayscale analysis.

### 2.5. Muscle Strength

Isometric knee extensor, ankle dorsiflexor, and ankle plantar flexor strength was assessed using a Lafayette model 01165A hand-held dynamometer (Lafayette Instrument Company, Lafayette, IN, USA). Readings recorded in kilogram-force were converted to newtons using 1 kgf = 9.80665 N. Individual moment-arm distances were not recorded; therefore, joint torque and body-mass-normalized torque could not be calculated. Hand-held dynamometry has demonstrated acceptable reliability for lower-limb strength assessment [26,27]. All strength assessments were performed by the same male physiatrist, who had four years of experience in muscle strength assessment and was blinded to the ultrasound results. Stabilization differed by test. An adjustable stabilizing belt was used during knee extension testing. During ankle dorsiflexion and plantar flexion testing, the dynamometer was stabilized manually by the examiner, who used body weight and bracing against a fixed support surface to resist the participant’s force. No stabilizing belt was used during either ankle test. The examiner demonstrated each maneuver before testing. Each contraction lasted 5 s and was repeated three times with at least 90 s of rest between attempts. The highest value was selected because the measurement objective was to quantify the greatest voluntary isometric force achieved during testing. This choice is consistent with previous lower-limb dynamometry research using the highest force across repeated trials [28]. Only the maximum value for each movement and side was retained; individual trial values were not recorded separately. Consequently, sample-specific dynamometric reliability, standard error of measurement, and between-trial coefficients of variation could not be calculated. Standardized test positions are shown in Figure 4.

### 2.6. Statistical Analysis

The original statistical analyses were performed using IBM SPSS Statistics, version 26.0 (IBM Corp., Armonk, NY, USA). Additional analyses conducted during revision, including Bland–Altman plotting, were performed using Python 3.12.14, with NumPy 2.3.5 and SciPy 1.17.0 for numerical and statistical calculations and Matplotlib 3.10.8 for plotting. Normally distributed continuous variables are reported as mean ± standard deviation (SD), model-estimated outcomes as mean (standard error [SE]), skewed variables as median (interquartile range [IQR]), and categorical variables as *n* (%). Independent-samples *t*-tests or Mann–Whitney U-tests were used for continuous or ordinal baseline variables as appropriate, and Fisher’s exact test was used for categorical variables. Absolute effect sizes for baseline comparisons were reported using Cohen’s d for independent-samples *t*-tests, Rosenthal’s r (|Z|/√N, where N = 60) for Mann–Whitney U tests, Cramér’s V for education level, and the phi coefficient for limb dominance. Education level was compared using the Fisher–Freeman–Halton exact test, and limb dominance using Fisher’s exact test.

Bilateral muscle architecture and strength outcomes were compared using a two-way mixed analysis of variance with group (r-axSpA vs. control) as the between-participant factor and side (right vs. left) as the within-participant factor. Complete bilateral data were available for all 60 participants, with 30 participants in each group. Results are reported as group marginal means, between-group differences (r-axSpA minus control), 95% confidence intervals (CIs), partial eta-squared (ηp^2^), and *p*-values. Two-sided 95% confidence intervals for partial eta-squared were obtained by inversion of the noncentral F distribution for the between-group effect (numerator df = 1; denominator df = 58), with noncentrality limits transformed to the effect-size scale using N = 60 participants. These calculations were performed in Python using SciPy. Within the r-axSpA group, an exploratory analysis compared biologic users and non-users with the same group-by-side framework.

An additional deterministic sensitivity analysis evaluated the effect of pennation-angle perturbations on derived fascicle length. Using the examiner-specific thickness and angle values available for each participant and side, fascicle length was recalculated as MT/sin(θ + δ), with δ set to −2°, −1°, +1°, and +2°, while holding muscle thickness constant. The same angular offset was applied to both groups. Recalculated lengths were averaged across examiners and sides for each participant. Between-group differences and 95% confidence intervals were calculated using an independent-samples *t* test with pooled variance, corresponding to the group main-effect comparison in the balanced group-by-side design. These scenarios assessed sensitivity to a common systematic angular offset and did not estimate actual measurement error.

Pearson or Spearman correlation coefficients, selected according to distributional assumptions, were used to examine associations between dominant-limb muscle architecture and strength in the full sample and associations with BASFI and BASMI. Correlations of 0.30–0.49, 0.50–0.69, and 0.70–0.89 were interpreted as weak, moderate, and strong, respectively [29].

Exploratory bivariate analyses within the r-axSpA group examined CRP in relation to dominant-limb architecture and strength. Variables associated with CRP were entered into separate multivariable linear regression models with CRP as the dependent variable and age, BMI, disease duration, BASDAI, and BASFI as covariates. Because only 30 patients contributed to models containing six predictors, these analyses were considered hypothesis-generating. Ultrasound reliability was assessed using the Reliability Analysis procedure in IBM SPSS Statistics version 26.0 (Analyze > Scale > Reliability Analysis), with a two-way mixed-effects model and absolute agreement. Both single-measure and average-measures intraclass correlation coefficients (ICCs) were calculated with 95% confidence intervals. Intraobserver analyses used the three measurements obtained from separately acquired images by each examiner (k = 3). Interobserver analyses compared the two examiner-specific means, each derived from three images independently acquired and measured by that examiner (k = 2). Thus, the single-measure interobserver ICC represents the reliability of one examiner’s three-image mean, whereas the average-measures ICC represents the reliability of the mean across both examiners. These interobserver estimates include acquisition and measurement variability but do not represent the reliability of a single ultrasound acquisition. ICC values <0.50, 0.50–0.74, 0.75–0.89, and ≥0.90 were considered poor, moderate, good, and excellent, respectively [30]. Bland–Altman plots were constructed separately for each muscle, side, and architectural parameter. Differences were calculated as examiner 2 minus examiner 1, and the estimated 95% limits of agreement were defined as the mean difference ± 1.96 standard deviations of the differences. All tests were two-sided, with *p* < 0.05 indicating statistical significance.

Sample size was determined a priori using G*Power version 3.1, with vastus lateralis muscle thickness designated as the primary outcome. The calculation used the “*t* tests” family and “Means: Difference between two independent means (two groups)” procedure, with a two-tailed test, an assumed Cohen’s d of 0.80, an α level of 0.05, a power of 0.80, and an allocation ratio of 1:1. The assumed effect size represented Cohen’s conventional large effect benchmark [31] rather than an estimate derived from a previous study or pilot data. The calculation required 26 participants per group; 30 participants per group were included. This calculation addressed the primary between-group comparison and did not establish adequate power for interaction, subgroup, or multivariable analyses.

## 3. Results

All 60 enrolled participants (30 r-axSpA and 30 controls) completed the assessments and were included in the analysis (Figure 1). The combined mean age was 44.0 ± 8.9 years. Age, height, weight, BMI, and limb dominance did not differ between groups. Educational attainment was higher among controls (*p* < 0.001). No included patient had hip joint involvement or peripheral arthritis. Clinical and ultrasound assessment identified no enthesitis or enthesopathic changes at the Achilles or patellar tendon insertions. Twenty-five patients (83.3%) were HLA-B27 positive. Radiographic sacroiliitis was bilateral grade 2 in 16 patients (53.3%), unilateral grade 3 in six (20.0%), and bilateral grade 3 in eight (26.7%). Clinical and treatment characteristics of the r-axSpA group are shown in Table 1.

### 3.1. Muscle Architecture and Strength

No significant group-by-side interaction was observed for any architecture or strength outcome (all *p* ≥ 0.089); individual interaction *p*-values are reported in Table 2. Compared with controls, participants with r-axSpA had lower VL, GM, and GL thickness and pennation angles. Fascicle length did not differ for these three muscles. By contrast, TA superficial-region thickness and derived fascicle length were higher in r-axSpA, whereas superficial-region pennation angle did not differ. Knee extensor, ankle dorsiflexor, and ankle plantar flexor strength were all lower in the r-axSpA group.

In the angle-perturbation sensitivity analysis, the between-group difference in derived TA superficial-region fascicle length remained positive across all scenarios, ranging from 2.09 cm with a +2° offset (95% CI 1.19–2.99) to 2.85 cm with a −2° offset (95% CI 1.60–4.10; all *p* < 0.001). Between-group differences in derived VL, GM, and GL fascicle lengths remained nonsignificant across all scenarios.

### 3.2. Correlations, Exploratory Regression, and Reliability

The associations summarized in Table 3 ranged from very weak to moderate in magnitude. The correlation between VL pennation angle and knee extensor strength was statistically significant but very weak (r = 0.280, *p* = 0.031), falling below the specified weak-correlation range of 0.30–0.49. VL thickness was positively correlated with knee extensor strength. GM thickness and pennation angle and GL thickness and pennation angle were positively correlated with ankle plantar flexor strength. TA superficial-region thickness and derived fascicle length were inversely correlated with ankle dorsiflexor strength. Correlations with BASFI (r = −0.27 to 0.22) and BASMI (r = −0.17 to 0.19) were not statistically significant.

Within the r-axSpA group, CRP was positively correlated with dominant-limb GM pennation angle (r = 0.522, *p* = 0.003). An inverse correlation was also observed between CRP and derived dominant-limb GM fascicle length (r = −0.386, *p* = 0.035). Because fascicle length was calculated as muscle thickness/sin(θ), these associations are mathematically coupled and should not be interpreted as independent findings. In the adjusted model for GM pennation angle, a one-degree-higher angle was associated with a 1.256 mg/L higher CRP level (95% CI 0.394–2.119; *p* = 0.006; Table 4). The overall model was statistically significant (R^2^ = 0.423; adjusted R^2^ = 0.273; F (6,23) = 2.811; *p* = 0.034). The separate adjusted model for derived GM fascicle length was not significant (F (6,23) = 1.618; *p* = 0.187).

Inter-rater reliability estimates are presented in Table S2. Intraobserver ICCs were also excellent for both evaluators (Table S3).

## 4. Discussion

In this controlled cross-sectional study of men with r-axSpA, VL, GM, and GL thickness and pennation angles were lower than in age-matched healthy men, whereas TA superficial-region thickness and derived fascicle length were higher. Isometric strength was lower across knee extension, ankle dorsiflexion, and ankle plantar flexion. Architecture–strength correlations were modest, and BASFI and BASMI were not associated with the peripheral muscle outcomes. These results indicate that lower-limb architecture and voluntary strength capture related but non-equivalent aspects of muscle status in r-axSpA.

Most axSpA imaging studies have focused on the paraspinal muscles [6,7,8]. The lower VL, GM, and GL thickness observed here is compatible with reduced muscle size, while smaller pennation angles indicate altered fascicle arrangement. However, the present design cannot determine whether these differences reflect disease-related inflammation, lower habitual activity, pain-related inhibition, medication exposure, or other unmeasured factors. In rheumatoid arthritis, ultrasound-detected quadriceps abnormalities and longitudinal changes have similarly been linked with function or disease activity [16,17], supporting broader investigation of peripheral muscle phenotypes across inflammatory rheumatic diseases.

The TA findings deserve separate consideration. Despite substantially lower ankle dorsiflexor strength in r-axSpA (between-group difference, −52.0 N), TA superficial-region thickness and derived fascicle length were greater (+0.53 and +2.40 cm, respectively). These superficial-region parameters were also inversely correlated with dorsiflexor strength (Spearman’s ρ = −0.365 and −0.429, respectively). At the group level, greater TA superficial-region thickness and derived fascicle length co-occurred with lower dorsiflexion strength. This preliminary observation does not establish a physiological mechanism and requires confirmation in independent studies using validated measurement methods. These measurements do not represent full TA thickness or deep-region architecture and should not be interpreted as evidence of whole-muscle enlargement. Furthermore, the finding should not be labeled pseudo-hypertrophy based on the present evidence because echogenicity, intramuscular fat, edema, and cross-sectional area were not measured.

Reduced muscle quality is one possible explanation. MRI studies have documented paravertebral muscle atrophy and fatty degeneration in established ankylosing spondylitis and differences in paravertebral fat infiltration between non-radiographic axSpA and established disease [7,8]. These axial findings make fatty infiltration a biologically plausible hypothesis, but they cannot be extrapolated directly to the TA. Altered mechanical loading is another, non-exclusive possibility. Instrumented studies have identified lower walking speed, shorter step length, altered gait coordination, and other spatiotemporal or plantar-pressure abnormalities in ankylosing spondylitis [5,32]. One possible explanation is a compensatory gait pattern in which restricted spinal mobility and, when present, reduced hip excursion alter limb advancement during the swing phase. Increased reliance on ankle dorsiflexion to maintain foot clearance could modify habitual TA loading and potentially contribute to its architectural differences. However, the cited gait findings provide contextual support rather than direct evidence of increased TA recruitment. Although patients with hip joint involvement were excluded, dynamic hip excursion during walking was not assessed. Neither gait kinematics nor phase-specific TA activation was measured; therefore, increased dorsiflexor recruitment remains a hypothesis and cannot be inferred from greater TA superficial-region thickness or derived fascicle length alone. Pain during strength testing was also not measured, so pain-related inhibition remains another untested explanation for reduced force production. Future longitudinal studies combining quantitative ultrasound (including echo intensity and cross-sectional area), MRI-based muscle composition, gait analysis, surface electromyography, and dynamometry are required to determine whether the TA pattern reflects biomechanical adaptation, impaired muscle quality, or both.

The lower strength in all tested muscle groups is clinically relevant because knee extensors and ankle muscles contribute to transfers, gait, and balance. The correlations with architecture were nevertheless generally small in magnitude. In particular, the statistically significant correlation between VL pennation angle and knee extensor strength was very weak (r = 0.280), and its statistical significance should not be interpreted as evidence of a clinically meaningful association. Voluntary strength also depends on neural activation, pain, motivation, joint mechanics, and contractile quality, none of which is fully represented by thickness or pennation angle [33]. Similarly, the absence of significant correlations with BASFI and BASMI does not establish that ultrasound is more sensitive than these indices; rather, the measures appear to address different domains.

The supplementary comparison of biological-therapy users and non-users was exploratory and involved small, unequal subgroups (22 versus 8 participants; Table S4). Nonsignificant findings should not be interpreted as evidence of equivalence or absence of a treatment-associated difference. The non-randomized design, treatment heterogeneity, and absence of pretreatment measurements further preclude causal conclusions about treatment effects.

The positive association between GM pennation angle and CRP was statistically significant under conventional standard errors but was not significant when HC3 heteroscedasticity-robust standard errors were used in the additional analysis. The association was therefore sensitive to the method used to estimate uncertainty. Selection of muscle variables based on bivariate associations with CRP may have biased the regression estimates and produced overly optimistic *p*-values. The reported confidence intervals and *p*-values do not account for this selection process and should therefore be interpreted as exploratory. The small sample and potentially influential observations further limit the stability of these findings, which require independent replication. The inverse bivariate association between CRP and derived GM fascicle length may reflect mathematical coupling through the pennation-angle term in the calculation, together with variation in muscle thickness. It therefore does not provide independent corroboration of the CRP–pennation angle association. Although inflammation can affect skeletal-muscle turnover and repair [34,35,36], these cross-sectional findings do not establish that inflammation caused architectural remodeling.

Measurement reliability was generally high, consistent with standardized limb-muscle ultrasound protocols [10,11,15]. Single-measure inter-rater ICCs were lower than their average-measures counterparts, particularly for left GL fascicle length (0.884 versus 0.938), illustrating the distinction between the reliability of one examiner’s result and that of the average of the two examiner-specific means. These inter-rater estimates compare examiner-specific means from independently acquired image sets and therefore include acquisition and measurement variability. However, each examiner-specific mean was based on three images, so these estimates should not be interpreted as the reliability of a single ultrasound acquisition. Bland–Altman analysis additionally quantified agreement in the original measurement units (Figures S1–S4); for left GL fascicle length, the estimated limits of agreement extended from −1.429 to 1.864 cm. Therefore, high ICCs alone should not be taken to establish a clinically acceptable agreement. Although the probe was lifted and repositioned and a new image was acquired for each repeat, reliability was assessed within the same session at marked anatomical sites. These estimates therefore reflect within-session acquisition and measurement repeatability and do not establish between-session reproducibility. The same skin markings were retained for both examiners; therefore, inter-rater reliability was assessed under standardized site-localization conditions and does not capture variability arising from independent identification of anatomical measurement sites. Although both examiners were blinded to group allocation during acquisition and offline measurement, real-time image quality assessment involved operator judgment. Unsaved views and probe adjustments were not logged, preventing quantification of any between-group differences in the acquisition process. Blinding reduced the potential for bias but could not eliminate operator-dependent variability or potential acquisition-related selection bias. Although initial ultrasound settings were standardized, anatomy-dependent adjustments to depth, focal zones, gain, and TGC may have influenced aponeurosis and fascicle visibility and introduced additional operator-dependent variability. Furthermore, fascicle length was mathematically derived from thickness and pennation angle, so its reliability does not provide independent confirmation of measurement reproducibility. For derived fascicle length, high reliability does not establish absolute accuracy or exclude amplification of angular measurement error at small pennation angles. Although the between-group findings were maintained under common angular offsets of ±1° and ±2°, this analysis did not address random measurement error, differential error between groups, or uncertainty in muscle thickness. The absolute fascicle-length estimates should therefore be interpreted cautiously.

This study has several limitations. Its cross-sectional design prevents temporal or causal inference. The single-center sample was small and included only men with radiographic disease, limiting generalizability to women and non-radiographic axSpA. Patients with hip involvement or peripheral arthritis were excluded, and no Achilles or patellar insertion enthesitis or enthesopathic changes were detected clinically or by ultrasound. This reduces the likelihood that these conditions explain the observed muscle findings, but limits generalizability to patients with such manifestations. Furthermore, spinal structural damage was not quantified using mSASSS. The available sacroiliitis grades do not substitute for this assessment; therefore, the potential contribution of spinal structural damage to muscle architecture and strength could not be evaluated. Sacroiliitis grades were extracted from existing clinical radiology reports without independent study-specific re-reading, and inter-rater reliability of radiographic grading was not assessed. Healthy controls differed in educational attainment, and habitual physical activity, nutrition, pain during testing, and occupational loading were not quantified, leaving residual confounding. Although regular exercisers were excluded using a defined frequency and session-duration threshold, eligibility was assessed by self-report during an interview. This screening did not quantify total habitual physical activity or establish comparable activity levels between groups. Differences in occupational, transport-related, and other daily activities may therefore have contributed to the observed differences in muscle architecture and strength. Recruitment of controls through an outpatient-clinic advertisement may also have introduced volunteer selection bias. Pain intensity during dynamometry was not assessed using a standardized scale. Consequently, we could not determine whether pain limited maximal voluntary force production or contributed to the observed between-group differences in strength. Smoking history was recorded in the patient group but was not included in the adjusted analyses, leaving potential residual confounding. Ultrasound did not include echogenicity or cross-sectional area, and fascicle length was derived rather than directly measured. TA architectural assessment was restricted to the superficial region; therefore, the findings cannot establish whether similar differences were present in the deep region or across the whole muscle. Only four selected muscles were examined. Unmeasured synergists, including soleus and rectus femoris, also contribute to the tested joint actions; therefore, joint-level strength differences cannot be attributed exclusively to the architecture of the imaged muscles. Joint positioning is an additional consideration. Although the ankle position during VL and TA imaging was verified using a goniometer and maintained manually, no external fixation device was used for TA imaging. During GM and GL imaging, the foot hung freely beyond the table edge, which did not ensure an identical ankle angle across participants. Consequently, between-group differences in resting ankle position may have contributed to the observed gastrocnemius architectural differences and cannot be excluded as an alternative explanation. The sensitivity analysis using common angular offsets did not address this potential differential positioning between groups. Probe pressure was controlled qualitatively by visual monitoring rather than objectively quantified, so residual variability in tissue compression cannot be excluded. Strength was expressed as force rather than joint torque because individual moment-arm distances were not recorded. Consequently, differences in limb dimensions and mechanical leverage could not be accounted for, and body-mass-normalized torque could not be evaluated. Ankle dorsiflexion and plantar flexion were assessed with manual dynamometer stabilization rather than belt fixation. Although the examiner used body weight and bracing against a fixed support surface, the ability to resist the participant’s force may still have limited the recorded values, particularly during plantar flexion. This could have underestimated force in stronger participants and affected the magnitude of between-group differences; the extent of this potential bias could not be quantified. Sample-specific dynamometric reliability and measurement error could not be quantified because only the maximum value from three trials was retained for each movement and side. Individual trial values were unavailable for calculating ICCs, SEMs, or between-trial coefficients of variation. Published reliability estimates from other populations cannot substitute for these sample-specific assessments, and selecting the maximum does not eliminate measurement variability. No reference method such as MRI or DXA and no test–retest acquisition on separate days were available. The numerous outcomes and correlation tests were not corrected for multiplicity. The sample-size calculation assumed a conventionally large between-group effect rather than an outcome-specific empirical estimate and did not address the full group-by-side analysis. The study may therefore have had insufficient power to detect smaller differences or interaction effects. The biologic subgroup and multivariable regression analyses were underpowered and exploratory.

Strengths include the controlled design, bilateral evaluation of four functionally distinct lower-limb muscles, blinded ultrasound and strength assessments, repeated measurements, and reporting of both inter- and intraobserver reliability. The findings provide quantitative estimates that can inform adequately powered longitudinal and rehabilitation studies.

## 5. Conclusions

Men with r-axSpA had lower VL, GM, and GL thickness and pennation angles and lower knee and ankle isometric strength than age-matched healthy men. Greater TA superficial-region thickness and derived fascicle length co-occurred with lower ankle dorsiflexion strength. This preliminary observation does not establish an underlying physiological mechanism or characterize whole-muscle TA architecture. Interpretation is limited by the indirect estimation of fascicle length and the potential influence of examiner strength during manually stabilized ankle testing. Independent studies using validated region-specific ultrasound measurements and externally stabilized dynamometry are needed to confirm these findings before clinical applications can be considered.

## Figures and Tables

**Figure 1:**
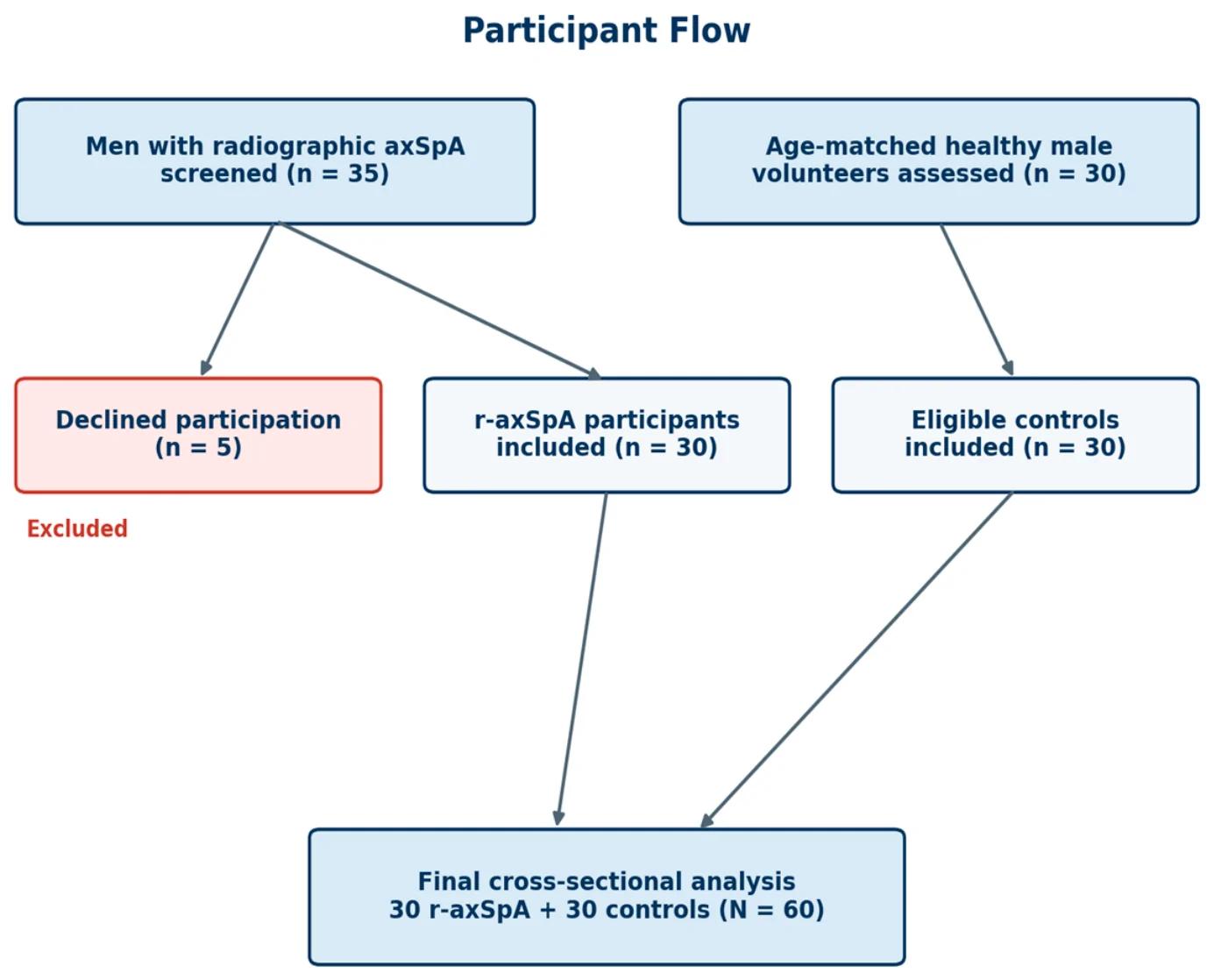
Participant flow through screening and analysis. r-axSpA, radiographic axial spondyloarthritis.

**Figure 2:**
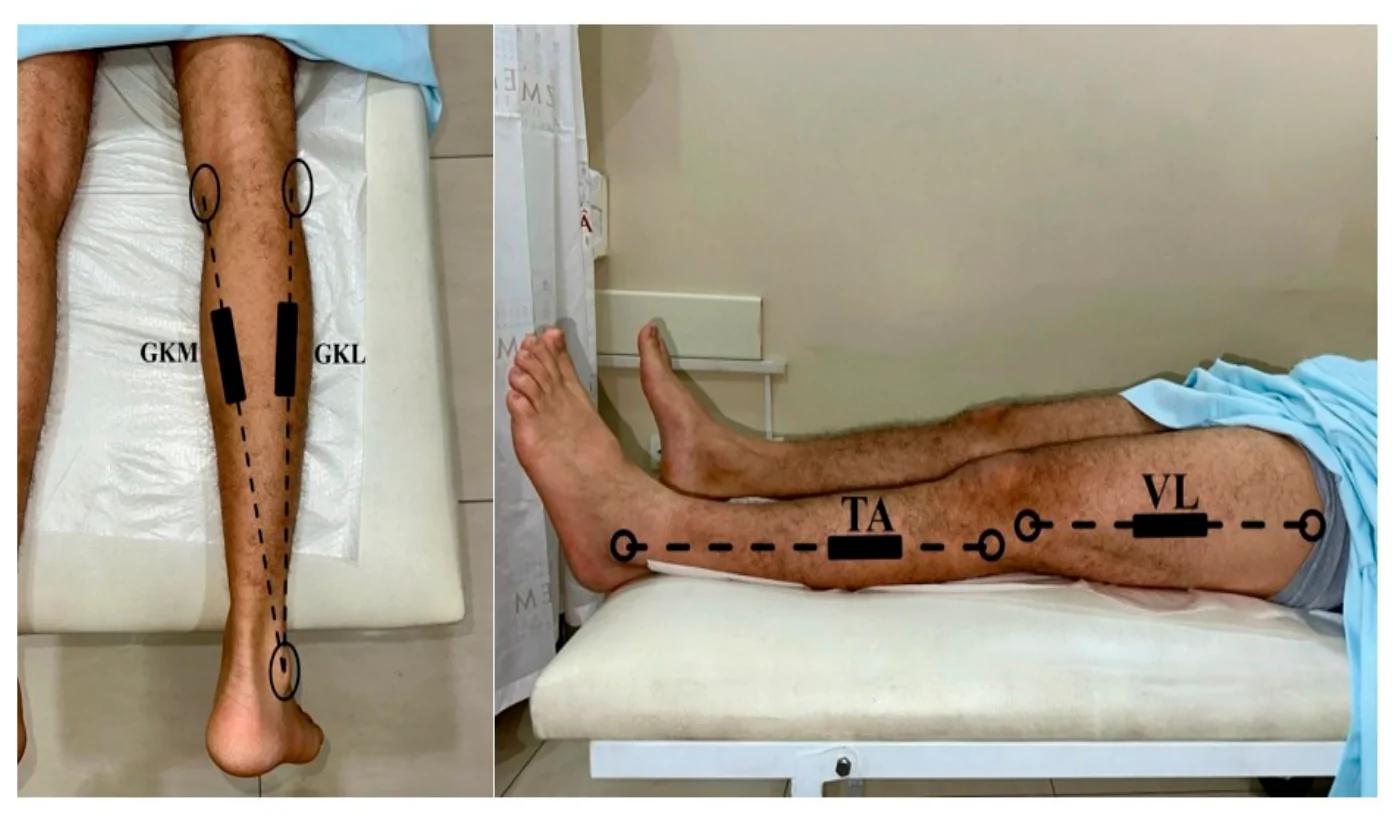
Standardized participant positions and probe locations for longitudinal ultrasound assessment of the gastrocnemius medialis, gastrocnemius lateralis, tibialis anterior, and vastus lateralis.

**Figure 3:**
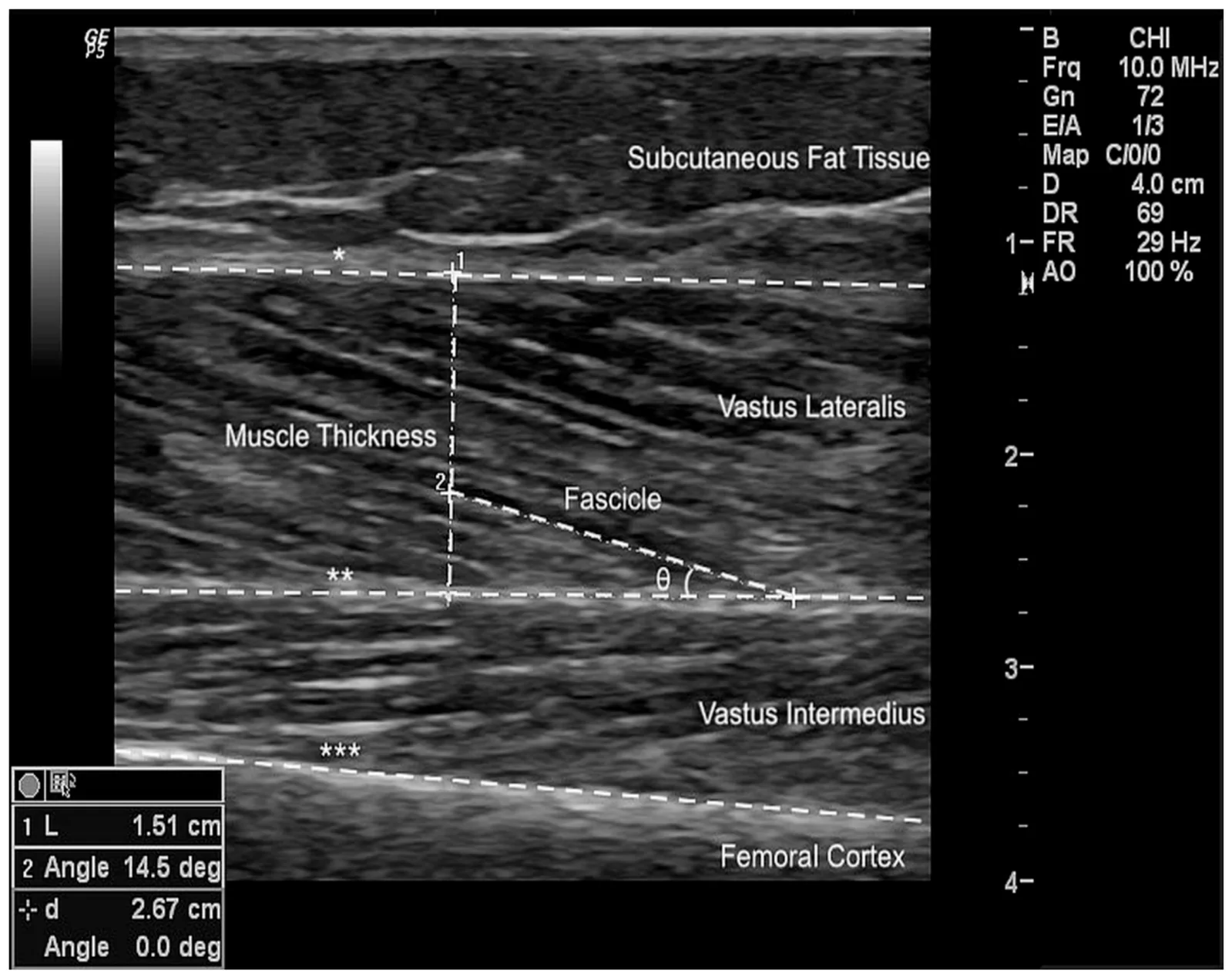
Longitudinal B-mode ultrasound image of the vastus lateralis. Muscle thickness was measured perpendicular to the superficial and deep aponeuroses, and pennation angle (θ) between the selected fascicle and deep aponeurosis. Fascicle length was not measured directly; it was derived as Lf = MT/sin(θ). The asterisks identify the superficial aponeurosis of VL (*), deep aponeurosis of VL (**), and femoral cortex (***). Horizontal dashed lines mark these anatomical boundaries; the vertical dashed line indicates the thickness measurement, and the oblique dashed line follows the selected fascicle.

**Figure 4:**
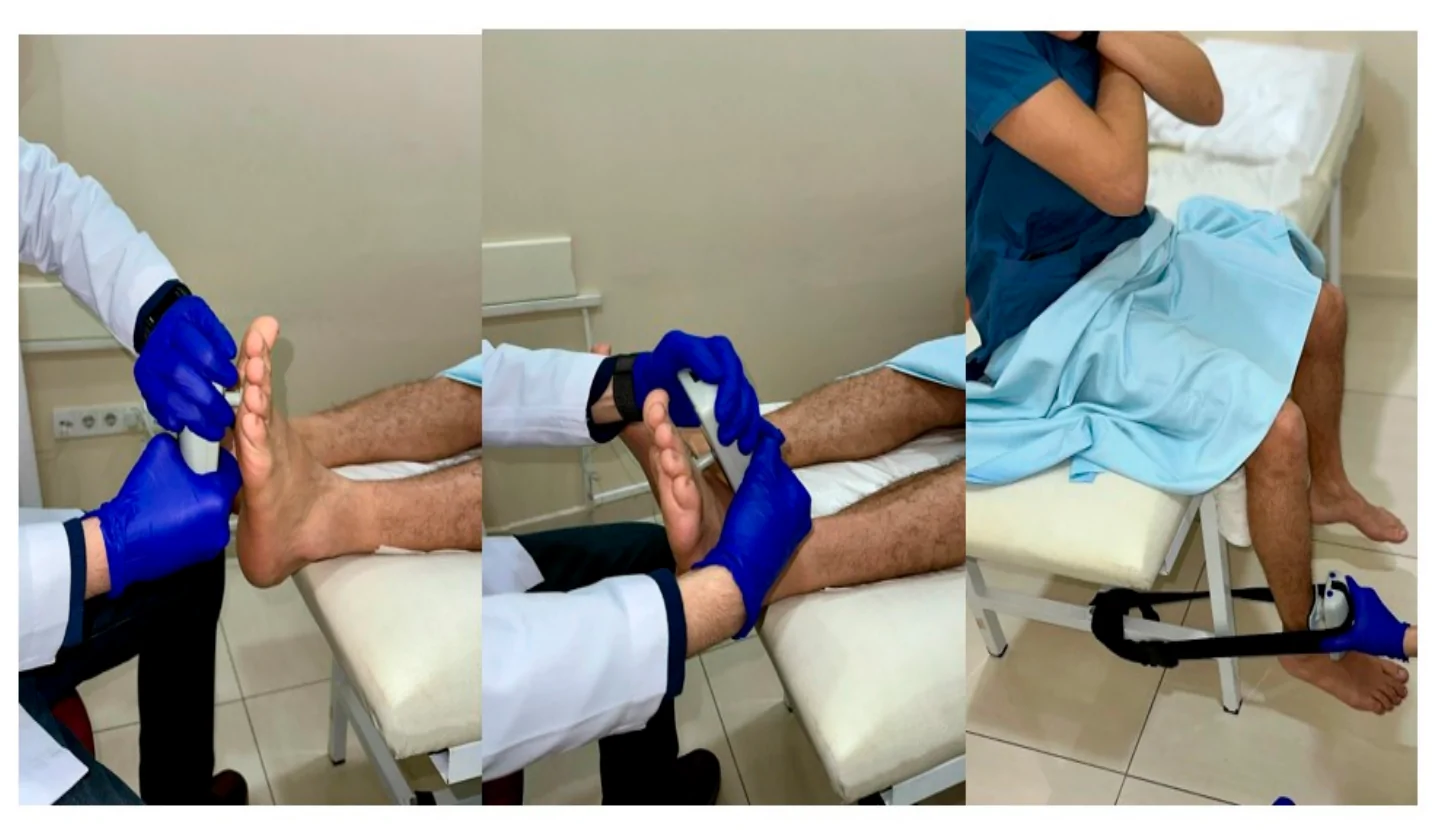
Test positions for isometric ankle plantar flexor, ankle dorsiflexor, and knee extensor strength assessment (left to right). An adjustable stabilizing belt was used during knee extension testing. Ankle plantar flexion and dorsiflexion were assessed with manual stabilization of the dynamometer by the examiner.

**Table 1:** Participant characteristics.

Characteristic	r-axSpA (*n* = 30)	Control (*n* = 30)	Statistical Test/Statistic	*p*-Value	Absolute Effect Size
**Demographic and anthropometric characteristics**					
Age (years)	43.9 ± 8.5	44.2 ± 9.4	t = −0.130	0.897	Cohen’s d = 0.033
Height (cm)	174.9 ± 5.5	176.6 ± 5.6	t = −1.186	0.241	Cohen’s d = 0.306
Weight (kg) *	78.0 (12.3)	80.0 (13.3)	Mann–Whitney U; |Z| = 0.399	0.690	Rosenthal’s r = 0.052
BMI (kg/m^2^) *	26.1 (3.1)	25.6 (4.5)	Mann–Whitney U; |Z| = 1.282	0.201	Rosenthal’s r = 0.165
**Education level,** ***n* (%)**			Fisher–Freeman–Halton exact test	<0.001	Cramér’s V = 0.510
Primary school	9 (30.0)	1 (3.3)	—	—	—
Middle school	1 (3.3)	2 (6.7)	—	—	—
High school	15 (50.0)	9 (30.0)	—	—	—
University	4 (13.3)	15 (50.0)	—	—	—
Master’s degree	1 (3.3)	3 (10.0)	—	—	—
**Limb dominance**					
Right-side dominance, *n* (%)	29 (96.7)	28 (93.3)	Fisher’s exact test	1.000	|φ| = 0.076
**Clinical and laboratory characteristics**					
Symptom duration (years)	11.7 ± 7.4	—	—	—	—
Time since diagnosis (years)	8.8 ± 6.4	—	—	—	—
BASDAI	3.6 ± 1.7	—	—	—	—
BASFI	2.0 ± 1.5	—	—	—	—
BASMI *	2.1 (0.8)	—	—	—	—
CRP (mg/L) *	4 (9)	—	—	—	—
HLA-B27 positive, *n* (%)	25 (83.3)	—	—	—	—
**Radiographic sacroiliitis,** ***n* (%)**			—		—
Bilateral grade 2	16 (53.3)	—	—	—	—
Unilateral grade 3	6 (20.0)	—	—	—	—
Bilateral grade 3	8 (26.7)	—	—	—	—
**Smoking status,** ***n*** **(%)**					
Never smoker	8 (26.7)	—	—	—	—
Former smoker	9 (30.0)	—	—	—	—
Current smoker	13 (43.3)	—	—	—	—
**Medication use and treatment duration**					
NSAID use, *n* (%)	10 (33.3)	—	—	—	—
Sulfasalazine use, *n* (%)	6 (20.0)	—	—	—	—
Anti-TNF use, *n* (%)	18 (60.0)	—	—	—	—
IL-17A inhibitor use, *n* (%)	4 (13.3)	—	—	—	—
Any biologic therapy, *n* (%)	22 (73.3)	—	—	—	—
Cumulative biologic treatment duration among users (months) *	42 (60)	—	—	—	—

Values are mean ± SD, median (IQR), or *n* (%), as appropriate. * Variables presented as median (IQR). Percentages are calculated within each group. The education *p*-value represents the overall between-group comparison using the Fisher–Freeman–Halton exact test. Limb dominance was compared using Fisher’s exact test. Medication categories are not mutually exclusive. Cumulative biologic treatment duration is reported for the 22 biologic therapy users. *p*-values are reported to three decimal places; values below 0.001 are reported as *p* < 0.001. Absolute effect sizes are reported: Cohen’s d for independent-samples *t*-tests, Rosenthal’s r (|Z|/√N, where N = 60) for Mann–Whitney U tests, Cramér’s V for education level, and the phi coefficient for limb dominance. Dashes indicate that data or comparisons are not applicable or not available. BASDAI, Bath Ankylosing Spondylitis Disease Activity Index; BASFI, Bath Ankylosing Spondylitis Functional Index; BASMI, Bath Ankylosing Spondylitis Metrology Index; BMI, body mass index; CRP, C-reactive protein; HLA-B27, human leukocyte antigen B27; IL-17A, interleukin-17A; IQR, interquartile range; NSAID, nonsteroidal anti-inflammatory drug; r-axSpA, radiographic axial spondyloarthritis; SD, standard deviation; TNF, tumor necrosis factor.

**Table 2:** Muscle architecture and isometric strength: group marginal means and between-group differences.

Outcome	r-axSpA Mean (SE)	Control Mean (SE)	Difference (95% CI)	ηp^2^ (95% CI)	Group *p*-Value	Group × Side *p*-Value
**Vastus lateralis (VL)**						
Muscle thickness (cm)	1.78 (0.06)	2.10 (0.06)	−0.32 (−0.50 to −0.13)	0.173 (0.032–0.337)	0.001	0.839
Pennation angle (°)	12.00 (0.38)	14.40 (0.38)	−2.38 (−3.48 to −1.28)	0.245 (0.075–0.408)	<0.001	0.089
Calculated fascicle length (cm)	8.70 (0.31)	8.50 (0.31)	0.12 (−0.77 to 1.03)	0.001 (0.000–0.069)	0.780	0.187
**Gastrocnemius medialis (GM)**						
Muscle thickness (cm)	1.50 (0.05)	1.70 (0.05)	−0.18 (−0.34 to −0.02)	0.084 (0.000–0.235)	0.025	0.498
Pennation angle (°)	14.60 (0.48)	16.60 (0.48)	−1.99 (−3.35 to −0.63)	0.129 (0.013–0.290)	0.005	0.729
Calculated fascicle length (cm)	6.20 (0.21)	6.00 (0.21)	0.16 (−0.44 to 0.77)	0.005 (0.000–0.092)	0.590	0.596
**Gastrocnemius lateralis (GL)**						
Muscle thickness (cm)	1.30 (0.05)	1.50 (0.05)	−0.21 (−0.36 to −0.05)	0.114 (0.008–0.273)	0.008	0.522
Pennation angle (°)	12.20 (0.43)	14.60 (0.43)	−2.46 (−3.69 to −1.23)	0.217 (0.057–0.381)	<0.001	0.433
Calculated fascicle length (cm)	6.40 (0.26)	6.21 (0.26)	0.15 (−0.57 to 0.89)	0.003 (0.000–0.084)	0.667	0.371
**Tibialis anterior (TA)**						
Superficial-region thickness (cm)	1.54 (0.08)	1.03 (0.08)	0.53 (0.29 to 0.78)	0.245 (0.075–0.409)	<0.001	0.515
Superficial-region pennation angle (°)	12.44 (0.33)	12.11 (0.33)	0.26 (−0.68 to 1.22)	0.005 (0.000–0.095)	0.577	0.623
Derived superficial-region fascicle length (cm)	7.42 (0.36)	5.03 (0.36)	2.40 (1.36 to 3.45)	0.269 (0.092–0.430)	<0.001	0.673
**Isometric strength**						
Knee extensor strength (N)	187.33 (13.12)	273.61 (13.11)	−86.31 (−123.62 to −48.83)	0.269 (0.092–0.430)	<0.001	0.992
Ankle dorsiflexor strength (N)	145.14 (9.12)	197.14 (9.12)	−52.01 (−78.21 to −26.63)	0.222 (0.060–0.386)	<0.001	0.720
Ankle plantar flexor strength (N)	176.51 (12.01)	234.44 (12.04)	−57.92 (−92.32 to −24.12)	0.168 (0.030–0.332)	0.001	0.449

VL, vastus lateralis; GM, gastrocnemius medialis; GL, gastrocnemius lateralis; TA, tibialis anterior; SE, standard error; CI, confidence interval; r-axSpA, radiographic axial spondyloarthritis; ANOVA, analysis of variance; ηp^2^, partial eta-squared; N, newton. Group values are estimated marginal means (SE), averaged across right and left sides. Between-group differences are calculated as r-axSpA minus control; positive values indicate higher values in the r-axSpA group. Outcomes were analysed using a two-way mixed ANOVA, with group as the between-participant factor and side as the within-participant factor. Group-effect and group-by-side interaction *p*-values are presented separately. Partial eta-squared and its 95% CI refer to the group effect. Fascicle length was calculated as Lf = MT/sin(θ), where Lf is fascicle length, MT is muscle thickness, and θ is pennation angle. For TA, all architectural parameters refer to the superficial region between the superficial and central aponeuroses; MT represents the thickness of this region. Strength is expressed in newtons (1 kgf = 9.80665 N). *p*-values are reported to three decimal places; values below 0.001 are reported as *p* < 0.001. Estimated marginal means, standard errors, between-group differences, and confidence limits for these differences are displayed to two decimal places. Partial eta-squared estimates and their confidence limits are displayed to three decimal places.

**Table 3:** Selected correlations between muscle architecture and muscle strength.

Strength Outcome	Architecture Parameter	Correlation Method	Correlation Coefficient	*p*-Value
Knee extensors	VL thickness	Pearson	0.420	<0.001
Knee extensors	VL pennation angle	Pearson	0.280	0.031
Ankle plantar flexors	GM thickness	Pearson	0.465	<0.001
Ankle plantar flexors	GM pennation angle	Pearson	0.354	0.005
Ankle plantar flexors	GL thickness	Pearson	0.539	<0.001
Ankle plantar flexors	GL pennation angle	Pearson	0.355	0.005
Ankle dorsiflexors	TA superficial-region thickness	Spearman	−0.365	0.004
Ankle dorsiflexors	TA derived superficial-region fascicle length	Spearman	−0.429	<0.001

Selected associations are summarized here. The correlation method is specified for each association: r denotes Pearson’s correlation coefficient, and ρ denotes Spearman’s rank correlation coefficient. The complete muscle architecture–strength correlation matrices for all 60 participants are provided in Supplementary Table S1, with Pearson correlations in Panel A and Spearman correlations in Panel B. Both panels include nonsignificant results, coefficients, and two-sided *p*-values. TA architectural parameters refer to the superficial region. GL, gastrocnemius lateralis; GM, gastrocnemius medialis; TA, tibialis anterior; VL, vastus lateralis.

**Table 4:** Exploratory multivariable linear regression of serum CRP in the r-axSpA group (*n* = 30).

Term	B	SE	Standardized β	*p*-Value	95% CI for B
Intercept	−15.751	10.470	—	0.146	−37.410 to 5.907
GM pennation angle, °	1.256	0.417	0.519	0.006	0.394 to 2.119
Age, years	0.100	0.109	0.167	0.369	−0.126 to 0.327
BMI, kg/m^2^	−0.099	0.336	−0.062	0.770	−0.794 to 0.595
BASDAI	−0.299	0.565	−0.104	0.602	−1.467 to 0.870
BASFI	0.226	0.678	0.070	0.742	−1.177 to 1.629
Disease duration, years	0.243	0.170	0.302	0.168	−0.110 to 0.595

Dependent variable: CRP (mg/L). R^2^ = 0.423; adjusted R^2^ = 0.273; F (6,23) = 2.811; *p* = 0.034. Owing to the small sample relative to the number of predictors and the skewed distribution of CRP, this analysis is hypothesis-generating. BASDAI, Bath Ankylosing Spondylitis Disease Activity Index; BASFI, Bath Ankylosing Spondylitis Functional Index; BMI, body mass index; CI, confidence interval; CRP, C-reactive protein; GM, gastrocnemius medialis; B, unstandardized regression coefficient; SE, standard error of the unstandardized regression coefficient.

## Data Availability

The data supporting the findings of this study are available from the corresponding author upon reasonable request, subject to ethics and privacy restrictions.

## Supplementary Materials

The following supporting information can be downloaded at: https://www.mdpi.com/article/10.3390/jcm15197515/s1. Table S1: Complete correlations between dominant-limb muscle architecture and isometric strength; Table S2: Interobserver reliability; Table S3: Intraobserver reliability; Table S4: Biologic-treatment subgroup analysis; Figure S1: Bland–Altman plots of inter-rater agreement for vastus lateralis (VL). Right and left limbs are shown in the left and right columns, respectively. Rows show muscle thickness, pennation angle, and fascicle length. Differences (examiner 2 minus examiner 1) are plotted against the mean of the two examiner-specific summary values. Solid lines indicate mean bias; dashed lines indicate the estimated 95% limits of agreement (mean difference ± 1.96 SD). Each point represents one participant; coincident points may overlap. Thickness and fascicle length are expressed in centimetres and pennation angle in degrees.; Figure S2: Bland–Altman plots of inter-rater agreement for medial gastrocnemius (GM). Right and left limbs are shown in the left and right columns, respectively. Rows show muscle thickness, pennation angle, and fascicle length. Differences (examiner 2 minus examiner 1) are plotted against the mean of the two examiner-specific summary values. Solid lines indicate mean bias; dashed lines indicate the estimated 95% limits of agreement (mean difference ± 1.96 SD). Each point represents one participant; coincident points may overlap. Thickness and fascicle length are expressed in centimetres and pennation angle in degrees; Figure S3: Bland–Altman plots of inter-rater agreement for lateral gastrocnemius (GL). Right and left limbs are shown in the left and right columns, respectively. Rows show muscle thickness, pennation angle, and fascicle length. Differences (examiner 2 minus examiner 1) are plotted against the mean of the two examiner-specific summary values. Solid lines indicate mean bias; dashed lines indicate the estimated 95% limits of agreement (mean difference ± 1.96 SD). Each point represents one participant; coincident points may overlap. Thickness and fascicle length are expressed in centimetres and pennation angle in degrees; Figure S4: Bland–Altman plots of inter-rater agreement for the superficial region of tibialis anterior (TA). Right and left limbs are shown in the left and right columns, respectively. Rows show superficial-region thickness, superficial-region pennation angle, and derived superficial-region fascicle length. Differences (examiner 2 minus examiner 1) are plotted against the mean of the two examiner-specific summary values. Solid lines indicate mean bias; dashed lines indicate the estimated 95% limits of agreement (mean difference ± 1.96 SD). Each point represents one participant; coincident points may overlap. Thickness and fascicle length are expressed in centimetres and pennation angle in degrees.

## Abbreviations

The following abbreviations are used in this manuscript:

axSpA	Axial spondyloarthritis
BASDAI	Bath Ankylosing Spondylitis Disease Activity Index
BASFI	Bath Ankylosing Spondylitis Functional Index
BASMI	Bath Ankylosing Spondylitis Metrology Index
BMI	Body mass index
CI	Confidence interval
CRP	C-reactive protein
DXA	Dual-energy X-ray absorptiometry
GL	Gastrocnemius lateralis
GM	Gastrocnemius medialis
ICC	Intraclass correlation coefficient
IL-17A	Interleukin-17A
IQR	Interquartile range
Lf	Fascicle length
MRI	Magnetic resonance imaging
MT	Muscle thickness
NSAID	Nonsteroidal anti-inflammatory drug
r-axSpA	Radiographic axial spondyloarthritis
SD	Standard deviation
SE	Standard error
SPSS	Statistical Package for the Social Sciences
STROBE	Strengthening the Reporting of Observational Studies in Epidemiology
TA	Tibialis anterior
TNF	Tumor necrosis factor
VL	Vastus lateralis
