# Supplementary material for: Lower-Limb Muscle Architecture and Strength in Men with Radiographic Axial Spondyloarthritis: A Cross-Sectional Ultrasound Study
Source: J Clin Med. 2026 Sep 27;15(19):7515. doi: 10.3390/jcm15197515 (PMC13666262; doi:10.3390/jcm15197515)

# Supplementary Material

## Lower-Limb Muscle Architecture and Strength in Men with Radiographic Axial Spondyloarthritis: A Cross-Sectional Ultrasound Study

Mehmet Serkan Kılıçoğlu et al.

# Supplementary Table S1

## Complete correlations between dominant-limb muscle architecture and isometric strength

### Panel A Pearson correlations

Full sample: 30 men with r-axSpA and 30 healthy controls. All cells use n = 60.

| Muscle architecture | Knee extension | | Ankle dorsiflexion | | Ankle plantar flexion | |
|---|---|---|---|---|---|---|
| | r | p | r | p | r | p |
| VL thickness | 0.420 | <0.001 | 0.334 | 0.009 | 0.392 | 0.002 |
| VL pennation angle | 0.280 | 0.031 | 0.474 | <0.001 | 0.499 | <0.001 |
| VL derived fascicle length | 0.188 | 0.149 | -0.078 | 0.553 | -0.039 | 0.766 |
| GM thickness | 0.386 | 0.002 | 0.441 | <0.001 | 0.465 | <0.001 |
| GM pennation angle | 0.404 | 0.001 | 0.294 | 0.022 | 0.354 | 0.005 |
| GM derived fascicle length | 0.066 | 0.617 | 0.171 | 0.192 | 0.172 | 0.189 |
| GL thickness | 0.501 | <0.001 | 0.527 | <0.001 | 0.539 | <0.001 |
| GL pennation angle | 0.386 | 0.002 | 0.452 | <0.001 | 0.355 | 0.005 |
| GL derived fascicle length | 0.202 | 0.123 | 0.118 | 0.367 | 0.219 | 0.093 |
| TA superficial-region thickness | -0.323 | 0.012 | -0.421 | <0.001 | -0.340 | 0.008 |
| TA superficial-region pennation angle | -0.065 | 0.624 | 0.000 | 0.998 | -0.010 | 0.940 |
| TA derived superficial-region fascicle length | -0.322 | 0.012 | -0.469 | <0.001 | -0.374 | 0.003 |

Architecture values were averaged across the two examiners for each participant’s dominant limb. Strength was taken from the same limb. Both panels report all 36 architecture–strength combinations, including nonsignificant results. Pearson and Spearman coefficients are presented separately for transparency; they are alternative summaries of the same associations, not independent confirmation. All p-values are two-sided and unadjusted for multiple testing. Analyses are exploratory. Pooled correlations may reflect between-group differences and should not be interpreted as within-group associations.

Abbreviations: GL, gastrocnemius lateralis; GM, gastrocnemius medialis; r-axSpA, radiographic axial spondyloarthritis; TA, tibialis anterior; VL, vastus lateralis. Fascicle length was derived as muscle thickness/sin(pennation angle). For TA, thickness and pennation angle were measured in the superficial region bounded by the superficial and central aponeuroses, and derived fascicle length refers to that region.

## Complete correlations between dominant-limb muscle architecture and isometric strength

### Panel B Spearman rank correlations

Full sample: 30 men with r-axSpA and 30 healthy controls. All cells use n = 60.

| Muscle architecture | Knee extension | | Ankle dorsiflexion | | Ankle plantar flexion | |
|---|---|---|---|---|---|---|
| | ρ | p | ρ | p | ρ | p |
| VL thickness | 0.440 | <0.001 | 0.309 | 0.016 | 0.356 | 0.005 |
| VL pennation angle | 0.301 | 0.019 | 0.423 | <0.001 | 0.455 | <0.001 |
| VL derived fascicle length | 0.101 | 0.444 | -0.136 | 0.300 | -0.119 | 0.366 |
| GM thickness | 0.382 | 0.003 | 0.325 | 0.011 | 0.413 | 0.001 |
| GM pennation angle | 0.364 | 0.004 | 0.308 | 0.017 | 0.277 | 0.032 |
| GM derived fascicle length | 0.071 | 0.591 | 0.098 | 0.457 | 0.180 | 0.168 |
| GL thickness | 0.488 | <0.001 | 0.445 | <0.001 | 0.524 | <0.001 |
| GL pennation angle | 0.409 | 0.001 | 0.491 | <0.001 | 0.358 | 0.005 |
| GL derived fascicle length | 0.036 | 0.784 | -0.014 | 0.913 | 0.152 | 0.246 |
| TA superficial-region thickness | -0.211 | 0.106 | -0.365 | 0.004 | -0.315 | 0.014 |
| TA superficial-region pennation angle | 0.031 | 0.815 | 0.072 | 0.586 | 0.029 | 0.826 |
| TA derived superficial-region fascicle length | -0.269 | 0.038 | -0.429 | <0.001 | -0.350 | 0.006 |

Architecture values were averaged across the two examiners for each participant’s dominant limb. Strength was taken from the same limb. Both panels report all 36 architecture–strength combinations, including nonsignificant results. Pearson and Spearman coefficients are presented separately for transparency; they are alternative summaries of the same associations, not independent confirmation. All p-values are two-sided and unadjusted for multiple testing. Analyses are exploratory. Pooled correlations may reflect between-group differences and should not be interpreted as within-group associations.

Abbreviations: GL, gastrocnemius lateralis; GM, gastrocnemius medialis; r-axSpA, radiographic axial spondyloarthritis; TA, tibialis anterior; VL, vastus lateralis. Fascicle length was derived as muscle thickness/sin(pennation angle). For TA, thickness and pennation angle were measured in the superficial region bounded by the superficial and central aponeuroses, and derived fascicle length refers to that region.

## Table S2. Interobserver Reliability

Interobserver reliability of bilateral muscle-architecture measurements.

| Muscle / side | Parameter | Single-measure ICC (95% CI) | Average-measures ICC (95% CI), k = 2 |
|---|---|---|---|
| VL / Right | Thickness | 0.982 (0.963–0.991) | 0.991 (0.981–0.996) |
| VL / Right | Pennation angle | 0.932 (0.863–0.967) | 0.965 (0.926–0.983) |
| VL / Right | Fascicle length | 0.976 (0.951–0.988) | 0.988 (0.975–0.994) |
| VL / Left | Thickness | 0.984 (0.968–0.992) | 0.992 (0.984–0.996) |
| VL / Left | Pennation angle | 0.968 (0.934–0.985) | 0.984 (0.966–0.992) |
| VL / Left | Fascicle length | 0.982 (0.962–0.992) | 0.991 (0.981–0.996) |
| GM / Right | Thickness | 0.999 (0.999–1.000) | 1.000 (0.999–1.000) |
| GM / Right | Pennation angle | 0.939 (0.876–0.970) | 0.968 (0.934–0.985) |
| GM / Right | Fascicle length | 0.962 (0.922–0.982) | 0.981 (0.959–0.991) |
| GM / Left | Thickness | 0.997 (0.993–0.998) | 0.998 (0.997–0.999) |
| GM / Left | Pennation angle | 0.976 (0.951–0.989) | 0.988 (0.975–0.994) |
| GM / Left | Fascicle length | 0.982 (0.963–0.991) | 0.991 (0.981–0.996) |
| GL / Right | Thickness | 0.998 (0.996–0.999) | 0.999 (0.998–1.000) |
| GL / Right | Pennation angle | 0.965 (0.926–0.983) | 0.982 (0.962–0.992) |
| GL / Right | Fascicle length | 0.967 (0.932–0.984) | 0.983 (0.965–0.992) |
| GL / Left | Thickness | 0.997 (0.995–0.999) | 0.999 (0.997–0.999) |
| GL / Left | Pennation angle | 0.956 (0.907–0.979) | 0.978 (0.951–0.990) |
| GL / Left | Fascicle length | 0.884 (0.772–0.943) | 0.938 (0.871–0.971) |
| TA / Right | Superficial-region thickness | 0.999 (0.999–1.000) | 1.000 (0.999–1.000) |
| TA / Right | Superficial-region pennation angle | 0.974 (0.946–0.987) | 0.987 (0.972–0.994) |
| TA / Right | Derived superficial-region fascicle length | 0.987 (0.974–0.994) | 0.994 (0.987–0.997) |
| TA / Left | Superficial-region thickness | 0.999 (0.999–1.000) | 1.000 (0.999–1.000) |
| TA / Left | Superficial-region pennation angle | 0.977 (0.953–0.989) | 0.989 (0.976–0.995) |
| TA / Left | Derived superficial-region fascicle length | 0.995 (0.989–0.997) | 0.997 (0.994–0.999) |

*ICCs were calculated using a two-way mixed-effects model with absolute agreement. Each examiner independently acquired and measured three images per muscle and side. Interobserver analyses compared the two examiner-specific three-image means and therefore included both acquisition and measurement variability. Single-measure ICCs describe one examiner's three-image mean; average-measures ICCs describe the average of the two examiner-specific means (k = 2). These estimates do not represent the reliability of one ultrasound acquisition. The same skin markings were used by both examiners, so independent site-localization variability was not assessed. All TA parameters refer to the superficial region between the superficial and central aponeuroses. CI, confidence interval; GL, gastrocnemius lateralis; GM, gastrocnemius medialis; ICC, intraclass correlation coefficient; TA, tibialis anterior; VL, vastus lateralis.*

## Table S3. Intraobserver Reliability

Intraobserver reliability of repeated same-session ultrasound measurements for Evaluators 1 and 2.

| Muscle / side | Parameter | Evaluator 1: single ICC (95% CI) | Evaluator 1: average ICC (95% CI), k = 3 | Evaluator 2: single ICC (95% CI) | Evaluator 2: average ICC (95% CI), k = 3 |
|---|---|---|---|---|---|
| VL / Right | Thickness | 0.996 (0.993–0.998) | 0.999 (0.998–0.999) | 0.997 (0.993–0.999) | 0.999 (0.998–1.000) |
| VL / Right | Pennation angle | 0.989 (0.940–0.996) | 0.996 (0.979–0.999) | 0.997 (0.994–0.998) | 0.999 (0.998–0.999) |
| VL / Right | Fascicle length | 1.000 (0.998–1.000) | 1.000 (0.999–1.000) | 0.999 (0.998–0.999) | 1.000 (0.999–1.000) |
| VL / Left | Thickness | 0.995 (0.988–0.998) | 0.998 (0.996–0.999) | 0.996 (0.991–0.998) | 0.999 (0.997–0.999) |
| VL / Left | Pennation angle | 0.994 (0.976–0.998) | 0.998 (0.992–0.999) | 0.997 (0.994–0.999) | 0.999 (0.998–1.000) |
| VL / Left | Fascicle length | 1.000 (0.999–1.000) | 1.000 (1.000–1.000) | 0.999 (0.998–0.999) | 1.000 (0.999–1.000) |
| GM / Right | Thickness | 0.989 (0.893–0.997) | 0.996 (0.962–0.999) | 0.990 (0.980–0.995) | 0.997 (0.993–0.998) |
| GM / Right | Pennation angle | 0.964 (0.611–0.990) | 0.988 (0.825–0.997) | 0.995 (0.990–0.998) | 0.998 (0.997–0.999) |
| GM / Right | Fascicle length | 0.998 (0.984–0.999) | 0.999 (0.995–1.000) | 0.999 (0.998–0.999) | 1.000 (0.999–1.000) |
| GM / Left | Thickness | 0.987 (0.857–0.996) | 0.996 (0.947–0.999) | 0.984 (0.970–0.992) | 0.995 (0.990–0.997) |
| GM / Left | Pennation angle | 0.983 (0.792–0.995) | 0.994 (0.919–0.998) | 0.998 (0.995–0.999) | 0.999 (0.998–1.000) |
| GM / Left | Fascicle length | 0.998 (0.984–0.999) | 0.999 (0.995–1.000) | 0.999 (0.998–1.000) | 1.000 (0.999–1.000) |
| GL / Right | Thickness | 0.996 (0.986–0.998) | 0.999 (0.995–0.999) | 0.995 (0.975–0.998) | 0.998 (0.991–0.999) |
| GL / Right | Pennation angle | 0.993 (0.943–0.998) | 0.998 (0.980–0.999) | 0.997 (0.995–0.998) | 0.999 (0.998–0.999) |
| GL / Right | Fascicle length | 0.998 (0.986–1.000) | 0.999 (0.995–1.000) | 1.000 (0.999–1.000) | 1.000 (1.000–1.000) |
| GL / Left | Thickness | 0.995 (0.980–0.998) | 0.998 (0.993–0.999) | 0.994 (0.980–0.998) | 0.998 (0.993–0.999) |
| GL / Left | Pennation angle | 0.994 (0.938–0.998) | 0.998 (0.978–0.999) | 0.997 (0.994–0.998) | 0.999 (0.998–0.999) |
| GL / Left | Fascicle length | 0.999 (0.988–1.000) | 1.000 (0.996–1.000) | 1.000 (1.000–1.000) | 1.000 (1.000–1.000) |
| TA / Right | Superficial-region thickness | 0.999 (0.997–0.999) | 1.000 (0.999–1.000) | 0.957 (0.923–0.978) | 0.985 (0.973–0.992) |
| TA / Right | Superficial-region pennation angle | 0.997 (0.994–0.999) | 0.999 (0.998–1.000) | 0.997 (0.994–0.998) | 0.999 (0.998–0.999) |
| TA / Right | Derived superficial-region fascicle length | 1.000 (1.000–1.000) | 1.000 (1.000–1.000) | 1.000 (1.000–1.000) | 1.000 (1.000–1.000) |
| TA / Left | Superficial-region thickness | 0.999 (0.999–1.000) | 1.000 (1.000–1.000) | 0.998 (0.996–0.999) | 0.999 (0.999–1.000) |
| TA / Left | Superficial-region pennation angle | 0.997 (0.994–0.998) | 0.999 (0.998–0.999) | 0.995 (0.990–0.997) | 0.998 (0.997–0.999) |
| TA / Left | Derived superficial-region fascicle length | 1.000 (1.000–1.000) | 1.000 (1.000–1.000) | 1.000 (1.000–1.000) | 1.000 (1.000–1.000) |

*Each examiner's intraobserver ICCs were calculated from measurements on three separately acquired images per muscle and side. Each examiner acquired and measured their own image set; these were not repeated readings of a single stored image. ICCs were calculated using a two-way mixed-effects model with absolute agreement. Single-measure ICCs refer to one repeated measurement; average-measures ICCs refer to the average of three same-session measurements (k = 3), separately for each evaluator. Estimates and 95% confidence intervals are reported to three decimal places. All TA parameters refer to the superficial region between the superficial and central aponeuroses. CI, confidence interval; ICC, intraclass correlation coefficient.*

## Table S4. Biologic-Treatment Subgroup Analysis

Exploratory comparison of participants with r-axSpA receiving biologic therapy and those not receiving biologic therapy.

| Characteristic | Biologic users (n = 22) | Non-users (n = 8) | Test statistic |
|---|---|---|---|
| Age (years) | 43.4 (8.7) | 45.1 (8.5) | t(28) = 0.465; p = 0.646 |
| BMI (kg/m²) | 26.1 (3.3) | 26.0 (3.0) | t(28) = 0.045; p = 0.964 |
| Disease duration, years | 9.6 (6.9) | 6.5 (4.0) | t(28) = 1.192; p = 0.243 |
| BASDAI | 3.6 (1.9) | 3.6 (1.4) | t(28) = 0.020; p = 0.984 |
| BASFI | 2.3 (1.5) | 1.2 (1.2) | t(28) = 1.840; p = 0.076 |

Values are mean (SD). Independent-samples t-tests were used. BASDAI, Bath Ankylosing Spondylitis Disease Activity Index; BASFI, Bath Ankylosing Spondylitis Functional Index; BMI, body mass index; SD, standard deviation.

| Outcome | Users: right | Users: left | Non-users: right | Non-users: left | Group effect | Group × side |
|---|---|---|---|---|---|---|
| VL thickness (cm) | 1.88 (0.4) | 1.77 (0.4) | 1.73 (0.4) | 1.50 (0.2) | F = 1.043; p = 0.316 | F = 0.241; p = 0.627 |
| VL pennation angle (°) | 12.5 (2.1) | 12.3 (2.5) | 11.2 (1.7) | 11.0 (1.9) | F = 2.186; p = 0.150 | F = 0.006; p = 0.940 |
| VL fascicle length (cm) | 8.7 (2.0) | 8.5 (2.2) | 9.0 (2.3) | 8.6 (2.2) | F = 0.037; p = 0.850 | F = 0.156; p = 0.696 |
| GM thickness (cm) | 1.5 (0.3) | 1.5 (0.3) | 1.4 (0.2) | 1.5 (0.1) | F = 0.020; p = 0.889 | F = 1.367; p = 0.252 |
| GM pennation angle (°) | 13.9 (2.2) | 14.9 (3.4) | 14.7 (2.0) | 15.3 (3.3) | F = 0.363; p = 0.551 | F = 0.173; p = 0.681 |
| GM fascicle length (cm) | 6.4 (1.4) | 6.1 (1.5) | 5.9 (1.3) | 6.2 (1.4) | F = 0.117; p = 0.735 | F = 1.803; p = 0.190 |
| GL thickness (cm) | 1.3 (0.3) | 1.3 (0.3) | 1.2 (0.3) | 1.2 (0.2) | F = 0.570; p = 0.457 | F = 0.116; p = 0.736 |
| GL pennation angle (°) | 12.2 (2.3) | 12.0 (2.5) | 11.7 (2.4) | 12.9 (2.4) | F = 0.049; p = 0.826 | F = 2.460; p = 0.128 |
| GL fascicle length (cm) | 6.3 (1.3) | 6.7 (1.8) | 6.3 (1.8) | 5.7 (1.2) | F = 0.700; p = 0.410 | F = 1.708; p = 0.202 |
| TA superficial-region thickness (cm) | 1.5 (0.6) | 1.5 (0.7) | 1.6 (0.3) | 1.5 (0.4) | F = 0.023; p = 0.879 | F = 0.985; p = 0.330 |
| TA superficial-region pennation angle (°) | 12.7 (2.1) | 11.9 (1.8) | 12.3 (2.2) | 12.7 (2.0) | F = 0.103; p = 0.750 | F = 1.802; p = 0.190 |
| TA derived superficial-region fascicle length (cm) | 7.1 (2.4) | 7.6 (3.3) | 8.0 (2.0) | 7.0 (1.6) | F = 0.022; p = 0.883 | F = 4.014; p = 0.055 |
| Knee-extensor strength, N | 191.2 (52.0) | 183.4 (54.9) | 189.3 (46.1) | 181.4 (33.3) | F = 0.010; p = 0.920 | F = 0.001; p = 0.975 |
| Ankle-dorsiflexor strength, N | 151.0 (42.2) | 147.1 (39.2) | 136.3 (38.2) | 133.4 (23.5) | F = 0.838; p = 0.368 | F = 0.012; p = 0.915 |
| Ankle-plantar-flexor strength, N | 181.4 (59.8) | 176.5 (50.0) | 172.6 (70.6) | 163.8 (66.7) | F = 0.221; p = 0.642 | F = 0.150; p = 0.702 |

This exploratory comparison involved 22 biological-therapy users and eight non-users. Nonsignificant results do not establish equivalence or exclude clinically relevant differences. No causal inference regarding treatment effects is possible. Values are mean (SD). TA thickness represents the superficial region between the superficial and central aponeuroses; TA pennation angle and derived fascicle length refer to the same region. Two-way mixed ANOVA was used, with biologic-treatment group as the between-participant factor and side as the within-participant factor; group-effect and interaction tests have df = 1,28. GL, gastrocnemius lateralis; GM, gastrocnemius medialis; TA, tibialis anterior; VL, vastus lateralis.

Figure S1. Bland–Altman plots of inter-rater agreement for vastus lateralis (VL). Right and left limbs are shown in the left and right columns, respectively. Rows show muscle thickness, pennation angle, and fascicle length. Differences (examiner 2 minus examiner 1) are plotted against the mean of the two examiner-specific summary values. Solid lines indicate mean bias; dashed lines indicate the estimated 95% limits of agreement (mean difference ± 1.96 SD). Each point represents one participant; coincident points may overlap. Thickness and fascicle length are expressed in centimetres and pennation angle in degrees.

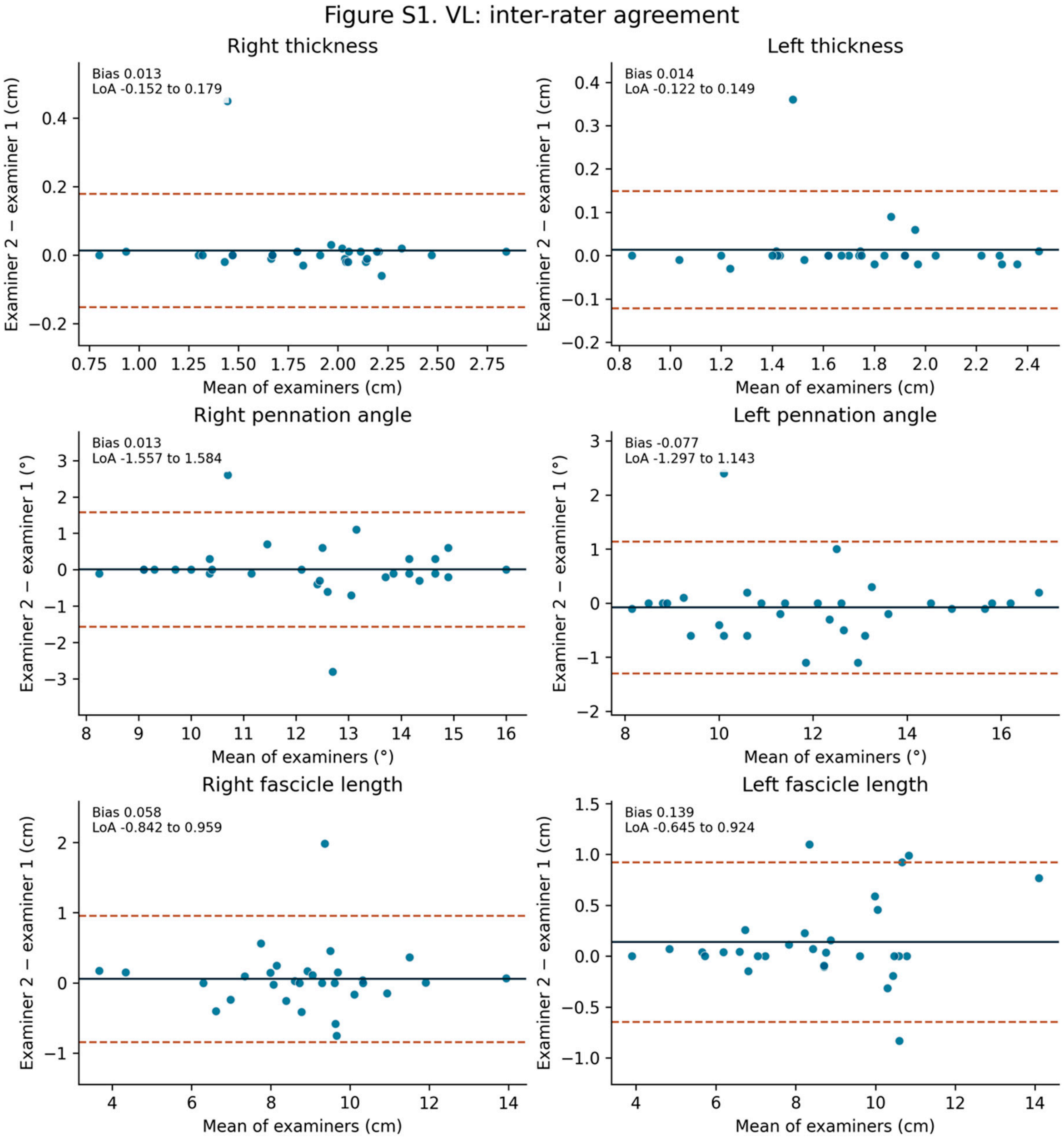

Figure S2. Bland–Altman plots of inter-rater agreement for medial gastrocnemius (GM). Right and left limbs are shown in the left and right columns, respectively. Rows show muscle thickness, pennation angle, and fascicle length. Differences (examiner 2 minus examiner 1) are plotted against the mean of the two examiner-specific summary values. Solid lines indicate mean bias; dashed lines indicate the estimated 95% limits of agreement (mean difference ± 1.96 SD). Each point represents one participant; coincident points may overlap. Thickness and fascicle length are expressed in centimetres and pennation angle in degrees.

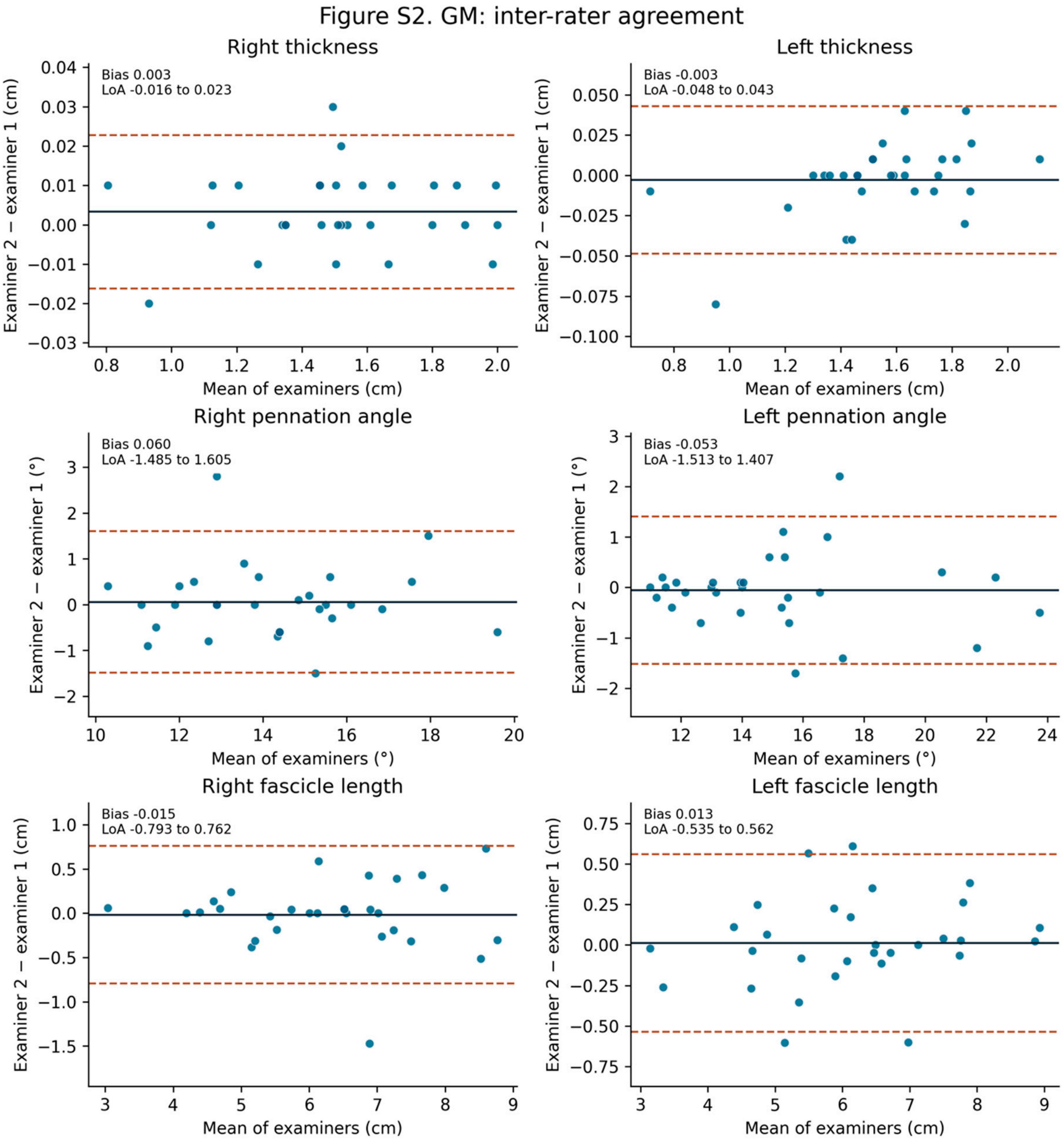

Figure S3. Bland–Altman plots of inter-rater agreement for lateral gastrocnemius (GL). Right and left limbs are shown in the left and right columns, respectively. Rows show muscle thickness, pennation angle, and fascicle length. Differences (examiner 2 minus examiner 1) are plotted against the mean of the two examiner-specific summary values. Solid lines indicate mean bias; dashed lines indicate the estimated 95% limits of agreement (mean difference ± 1.96 SD). Each point represents one participant; coincident points may overlap. Thickness and fascicle length are expressed in centimetres and pennation angle in degrees.

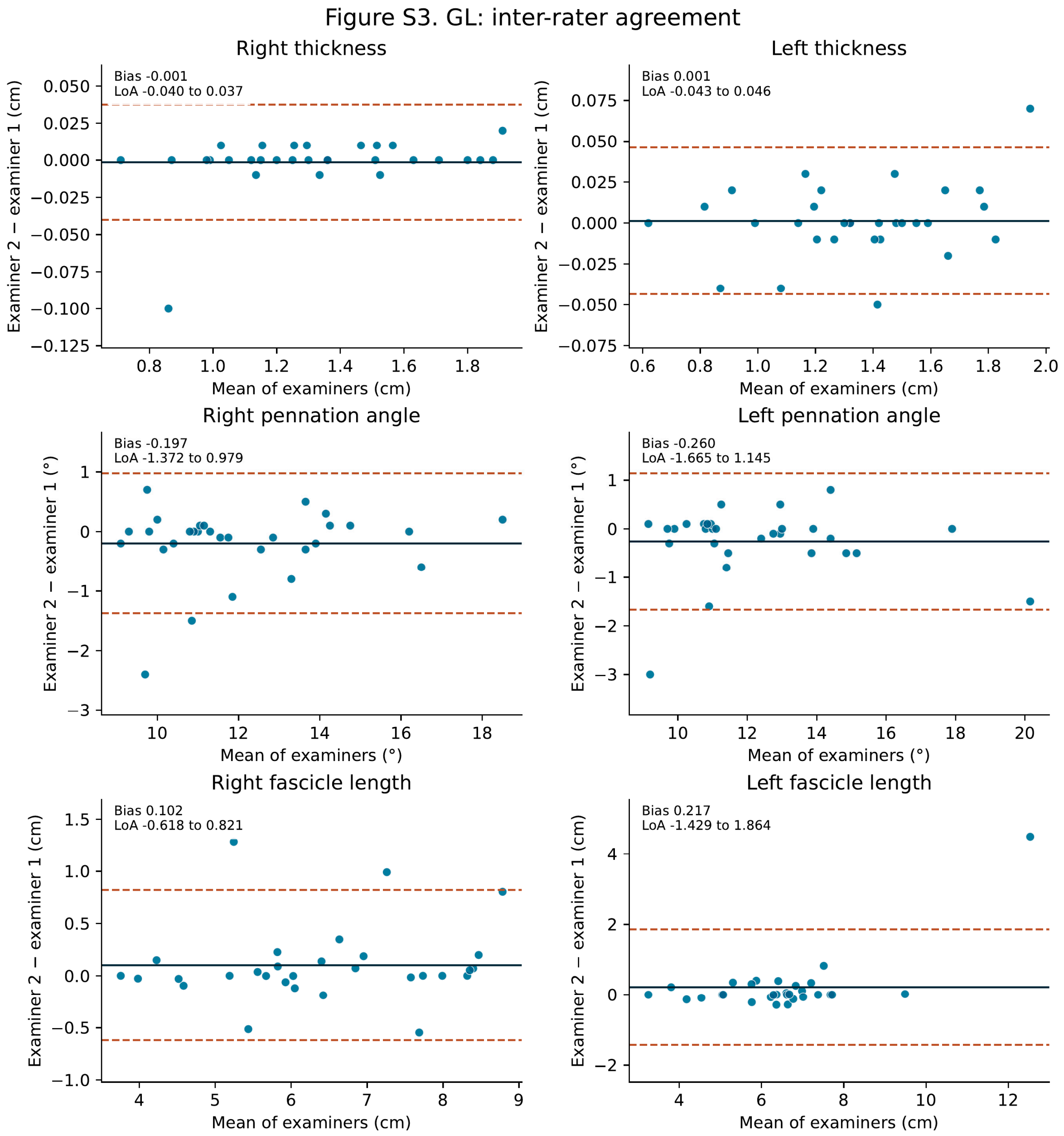

Figure S4. Bland–Altman plots of inter-rater agreement for the superficial region of tibialis anterior (TA). Right and left limbs are shown in the left and right columns, respectively. Rows show superficial-region thickness, superficial-region pennation angle, and derived superficial-region fascicle length. Differences (examiner 2 minus examiner 1) are plotted against the mean of the two examiner-specific summary values. Solid lines indicate mean bias; dashed lines indicate the estimated 95% limits of agreement (mean difference ± 1.96 SD). Each point represents one participant; coincident points may overlap. Thickness and fascicle length are expressed in centimetres and pennation angle in degrees.

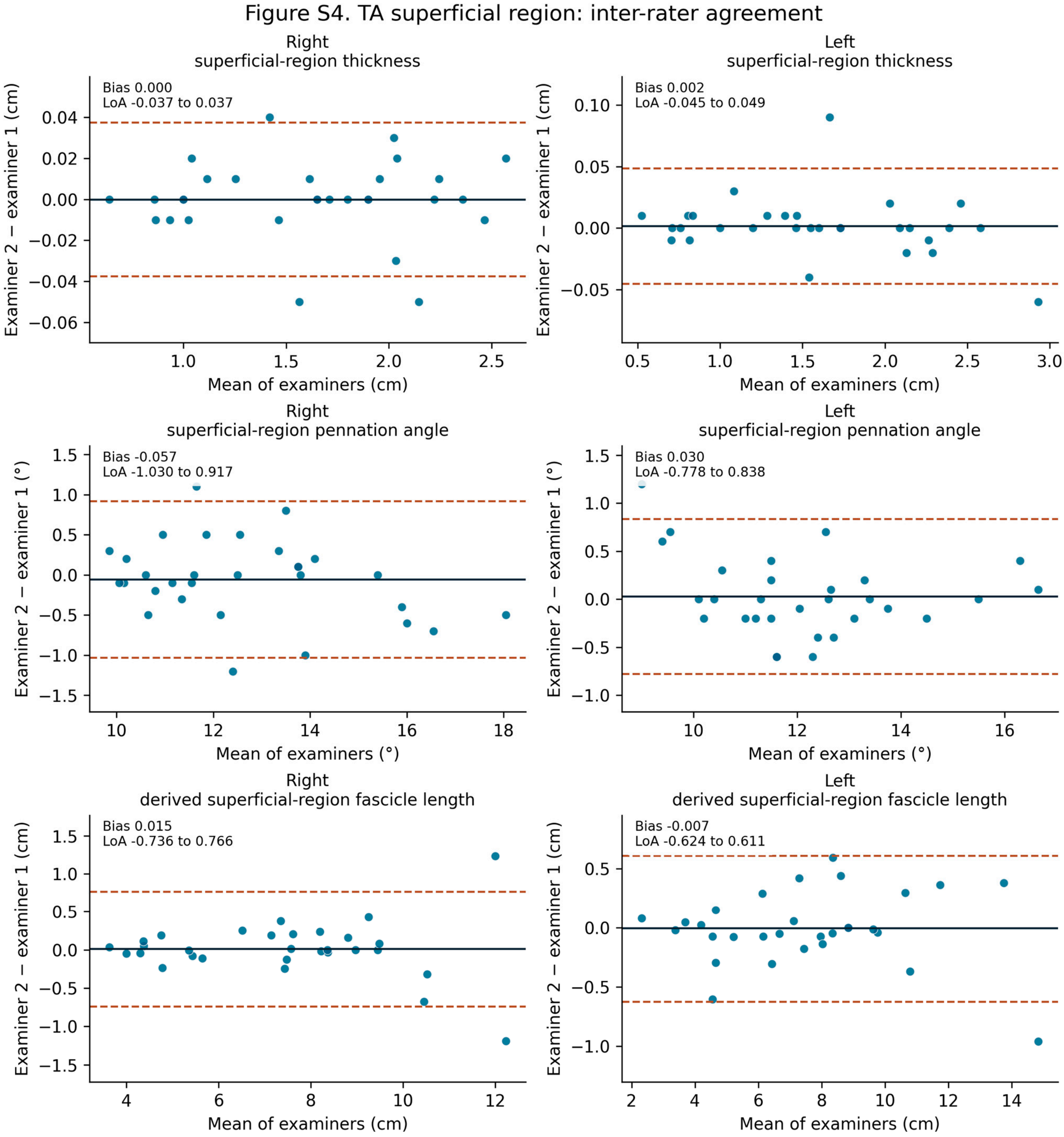

Supplement: Supplementary file 1 [file jcm-15-07515-s001.zip › jcm-4495675-supplementary.pdf]
